# PlaqView 2.0: A comprehensive web portal for cardiovascular single-cell genomics

**DOI:** 10.3389/fcvm.2022.969421

**Published:** 2022-08-08

**Authors:** Wei Feng Ma, Adam W. Turner, Christina Gancayco, Doris Wong, Yipei Song, Jose Verdezoto Mosquera, Gaëlle Auguste, Chani J. Hodonsky, Ajay Prabhakar, H. Atakan Ekiz, Sander W. van der Laan, Clint L. Miller

**Affiliations:** ^1^Medical Scientist Training Program, University of Virginia, Charlottesville, VA, United States; ^2^Center for Public Health Genomics, University of Virginia, Charlottesville, VA, United States; ^3^Research Computing, University of Virginia, Charlottesville, VA, United States; ^4^Department of Biochemistry and Molecular Genetics, University of Virginia, Charlottesville, VA, United States; ^5^Department of Computer Engineering, University of Virginia, Charlottesville, VA, United States; ^6^Department of Molecular Biology and Genetics, Izmir Institute of Technology, Gülbahçe, Turkey; ^7^Central Diagnostics Laboratory, Division Laboratories, Pharmacy, and Biomedical Genetics, University Medical Center Utrecht, Utrecht University, Utrecht, Netherlands; ^8^Department of Public Health Sciences, University of Virginia, Charlottesville, VA, United States

**Keywords:** scRNA-seq, single-cell, cardiovascular, genomics, web portal, database

## Abstract

Single-cell RNA-seq (scRNA-seq) is a powerful genomics technology to interrogate the cellular composition and behaviors of complex systems. While the number of scRNA-seq datasets and available computational analysis tools have grown exponentially, there are limited systematic data sharing strategies to allow rapid exploration and re-analysis of single-cell datasets, particularly in the cardiovascular field. We previously introduced PlaqView, an open-source web portal for the exploration and analysis of published atherosclerosis single-cell datasets. Now, we introduce PlaqView 2.0 (www.plaqview.com), which provides expanded features and functionalities as well as additional cardiovascular single-cell datasets. We showcase improved PlaqView functionality, backend data processing, user-interface, and capacity. PlaqView brings new or improved tools to explore scRNA-seq data, including gene query, metadata browser, cell identity prediction, *ad hoc* RNA-trajectory analysis, and drug-gene interaction prediction. PlaqView serves as one of the largest central repositories for cardiovascular single-cell datasets, which now includes data from human aortic aneurysm, gene-specific mouse knockouts, and healthy references. PlaqView 2.0 brings advanced tools and high-performance computing directly to users without the need for any programming knowledge. Lastly, we outline steps to generalize and repurpose PlaqView's framework for single-cell datasets from other fields.

## Introduction

Named “Method of the Year” in 2013, single-cell RNA sequencing (scRNA-seq) technology has now been used in virtually every field of biology and medicine, including cancer biology and cardiovascular medicine (1, 2). While scRNA-seq technologies continue to evolve and multi-modal measurements become increasingly more common, there has been a rapid increase in both the number of analysis tools and the complexity of single-cell data. At the time of writing, scrna-tools.org reports over 1,000 tools dedicated to single-cell data analysis (3). Despite the abundance of single-cell analysis tools available, there are two major challenges that affect the single-cell community as a whole: (1) there are no standardized methods of single-cell data sharing, and (2) increasingly complex and large single-cell data require advanced bioinformatic skills and resources for comprehensive analysis and interpretation.

The lack of standardized data sharing leads to the omission of critical metadata needed for reproducible analysis, such as author-defined cluster and cell-type annotations, and variables such as sex and age group (4). Despite the existence of public archives such as the Gene Expression Omnibus (GEO) and Sequence Read Archive (SRA), there is no uniform requirement for the deposition of metadata. Even within SRA, some datasets require specialized cloud computing tools (e.g., Google Cloud Computing) to access, such as the data from Li et al. (5). A recent study has found that < 50% of the published figures from public single-cell data can be reproduced as published (6). Furthermore, raw sequence files are very large (often >15 GB per file) and thus have very high computational resource requirements.

The fact that most tools require users to have significant programming skills remains a significant barrier to reanalyzing and exploring single-cell data, particularly across multiple datasets (7, 8). A few tools, such as the cBioPortal for Cancer Genomics (www.cbioportal.org), have distilled large bulk RNA-seq datasets, made them accessible to everyone, and provided multiple query functions, but none are designed for single-cell datasets (Supplemental Table 1) (9). Other portals, such as DISCO, provide real-time data integration and queries of multiple datasets across different tissues; however, DISCO currently lacks cardiovascular-specific datasets and comparison of analysis tools (Supplemental Table 1) (10). As more datasets emerge, the need for a centralized and domain-specific repository tailored for single-cell cardiovascular datasets becomes more pressing. A centralized repository packaged with tools specific for cardiovascular diseases will facilitate the advancement of the field as a whole and support the democratization and utility of single-cell data.

Previously, we introduced PlaqView, an open-source web portal focused on the exploration of the data generated by Wirka et al. (11) and a few other atherosclerosis-related datasets (12). Here, we introduce PlaqView 2.0, a significantly improved release with a broader scope to include, among others, datasets from other areas of the cardiovascular field, including human aortic aneurysm (13), healthy human heart atlas (14), human aortic valves (15), mouse models of atherogenesis (16), and others. We describe improvements to the user interface, new *ad hoc* functions to calculate trajectories on cells of interest, metadata exploration, and the ability to export publication-ready figures in addition to existing functions such as basic gene expression query and drug-gene interaction analysis. We also highlight several backend improvements that allow us to bring high-performance, reproducible computing environments to lay scientists *via* the web browser. Lastly, we outline basic steps to repurpose the PlaqView programming scaffold for other areas of single-cell investigation.

## Results

### PlaqView 2.0 includes significant expansion in data availability

**Figure 1:**
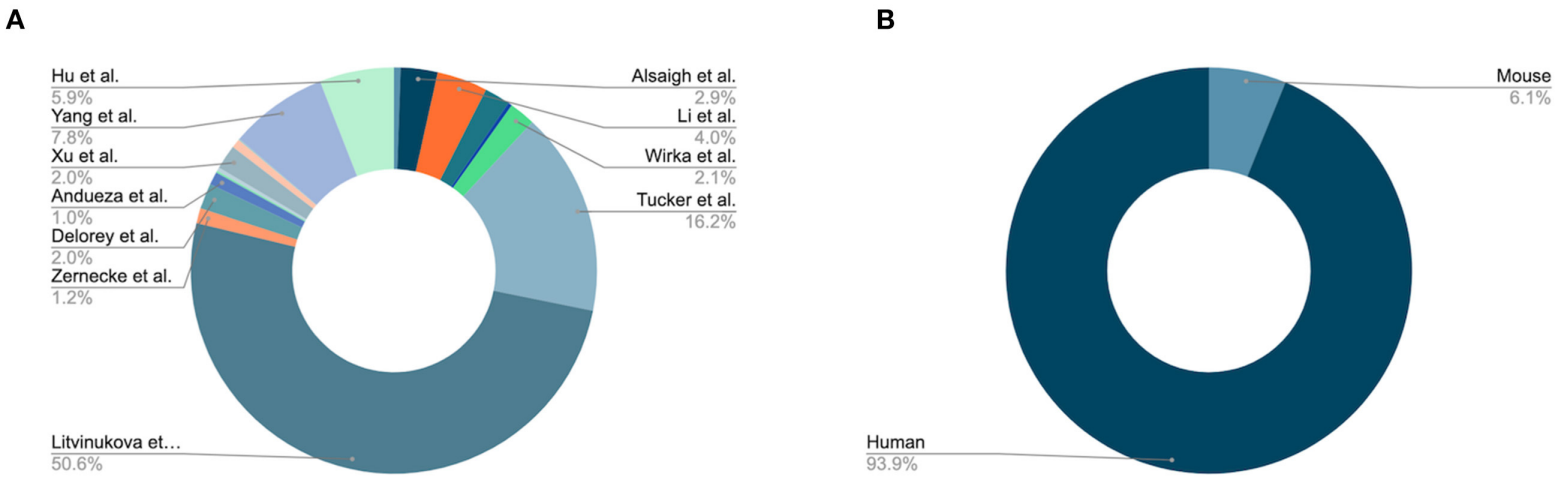
PlaqView 2.0 incorporates 32 cardiovascular-related datasets. **(A)** Major datasets available on PlaqView. PlaqView brings high-performance computing directly to the browser in order to handle large datasets such as ones from Litvinukova et al., which are atlas-type survey data that contain tissues from the entire human heart. **(B)** Species composition of PlaqView's database.

Since our initial publication introducing PlaqView, which featured 7 datasets from 4 studies (12), PlaqView 2.0 now features 32 datasets: 23 from human tissues and 9 from mouse tissues (Figure 1, Supplemental Table 2). New single-cell datasets made available in this release include those from: mouse carotid ligation experiments (17), mouse adventitial cell layer (18), human adult heart compartments (14), human COVID-19 autopsy hearts (19), and human aortic leaflets (15). At the time of writing, PlaqView now contains over 1.7 million total cells. PlaqView will be actively maintained and we will continue to add new datasets upon their publication and release to the public.

### PlaqView allows rapid query of gene expression of single cell datasets

**Figure 2:**
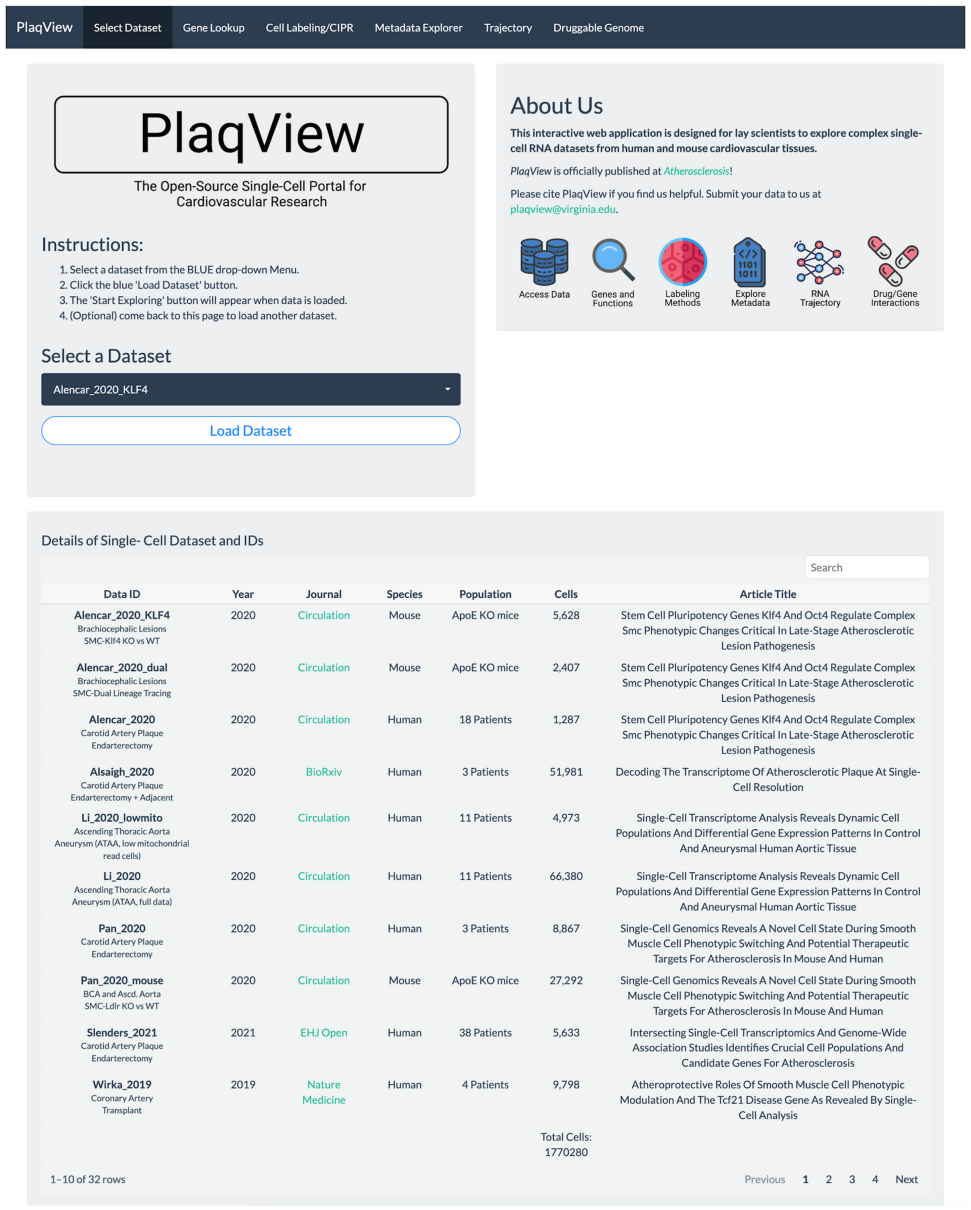
PlaqView 2.0 homepage facilitates selection and loading of relevant datasets. The homepage allows users to browse dataset details and load selected datasets into their dedicated session for further exploration.

**Figure 3:**
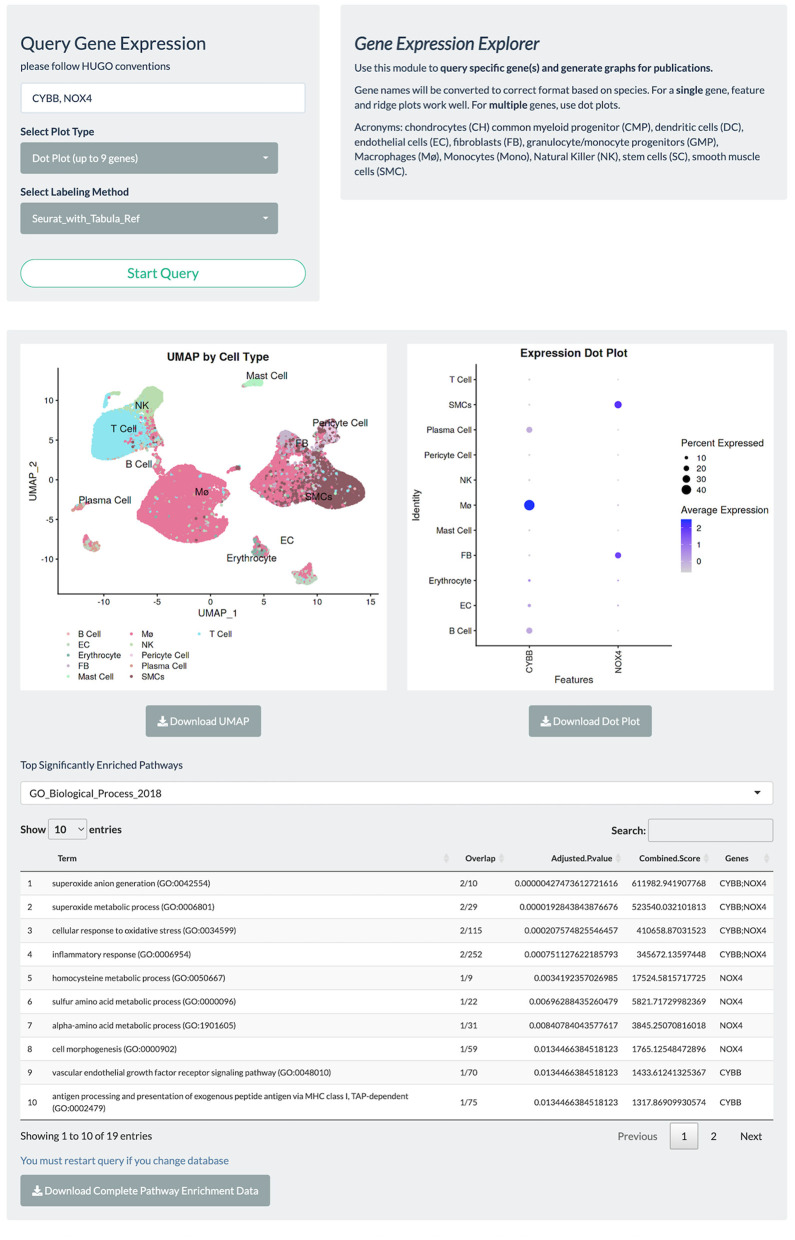
Gene query page allows rapid visualization of gene expression, UMAP embeddings, and facilitates automated GSEA. PlaqView 2.0 supports visualization of a single gene or multiple genes via feature, dot, and ridge plots, as powered by Seurat. Users can also choose their preferred annotation methods and download high-quality pdfs for publication. This instance is a demonstration using the Li et al. dataset.

Gene expression-based query is the mainstay for single-cell data exploration. However, this is also one of the most time-consuming steps because public data are often shared in various formats that require customized codes to be read and analyzed. In PlaqView 2.0, each dataset has been systematically preprocessed from various stages of upstream analysis to allow efficient querying on-demand. Upon launching the PlaqView homepage (www.plaqview.com), users can open the scRNA-seq portal (Figure 2), choose the scRNA-seq dataset of their interest, and load it into the memory of their dedicated session. Manually curated information about each dataset is also available on the portal and on the main homepage. Once the dataset is loaded, users are directed to the “Gene Lookup” tab (Figure 3) where they can enter a single gene or multiple genes, select the plot type they prefer (e.g., Dot Plot, Feature Plot, or Ridge Plot), and choose from one of the available cell-labeling methods (see next section) for query. The Gene Query function is powered by Seurat (7). Gene symbol capitalizations are automatically corrected based on species of the dataset selected to conform to Human Gene Nomenclature Committee (HGNC) or Mouse Genome Informatics (MGI) gene nomenclature conventions (e.g., *APOE* for humans and *Apoe* for mice) (20, 21). The queried gene(s) plots then will appear alongside the conventional UMAP displaying the cell type, and users can download high-quality, publication-ready figures in .pdf format.

To facilitate functional interpretation within a given single-cell dataset, PlaqView conducts automated Pathway Enrichment Analysis with the queried genes, powered by EnrichR (22–24). Although not commonly a part of a standard single-cell RNA-seq pipeline, pathway enrichments provide additional insights into the biological importance and functional consequences. Users can choose their preferred databases from a list of well-annotated sources such as ENCODE (Encyclopedia of DNA Elements) and GO (Gene Ontology). For example, querying the two NADPH oxidase genes implicated in redox metabolism and atherosclerosis “CYBB” and “NOX4” (25, 26), in the Li et al. (13) dataset using the “GO_Biological_Process_2018” database shows the top function category as “superoxide anion generation,” and that (FB), macrophages (Mø), and smooth muscle cells (SMCs) highly express these genes (Figure 3). This information allows users to quickly assess and generate hypotheses and aid in the design of future experiments.

### Cell labeling and differential gene expression

Cell identity prediction remains one of the most time-intensive and critical steps in the single-cell analysis pipeline (27). Previously, we found that the upstream cell-state prediction step greatly affects downstream analysis such as cell-cell communication analysis (12), and multiple labeling methods should be compared for consistency. Automated labeling tools are great starting points and help eliminate the inherent bias introduced in cluster-based manual annotation (27). However, current references may not accurately predict novel cell types or transitional states, such as the “myofibroblasts” as shown in Wirka et al. (11). Furthermore, discovery of novel niche cell-types are difficult and require careful examination of the differential gene expression patterns.

**Figure 4:**
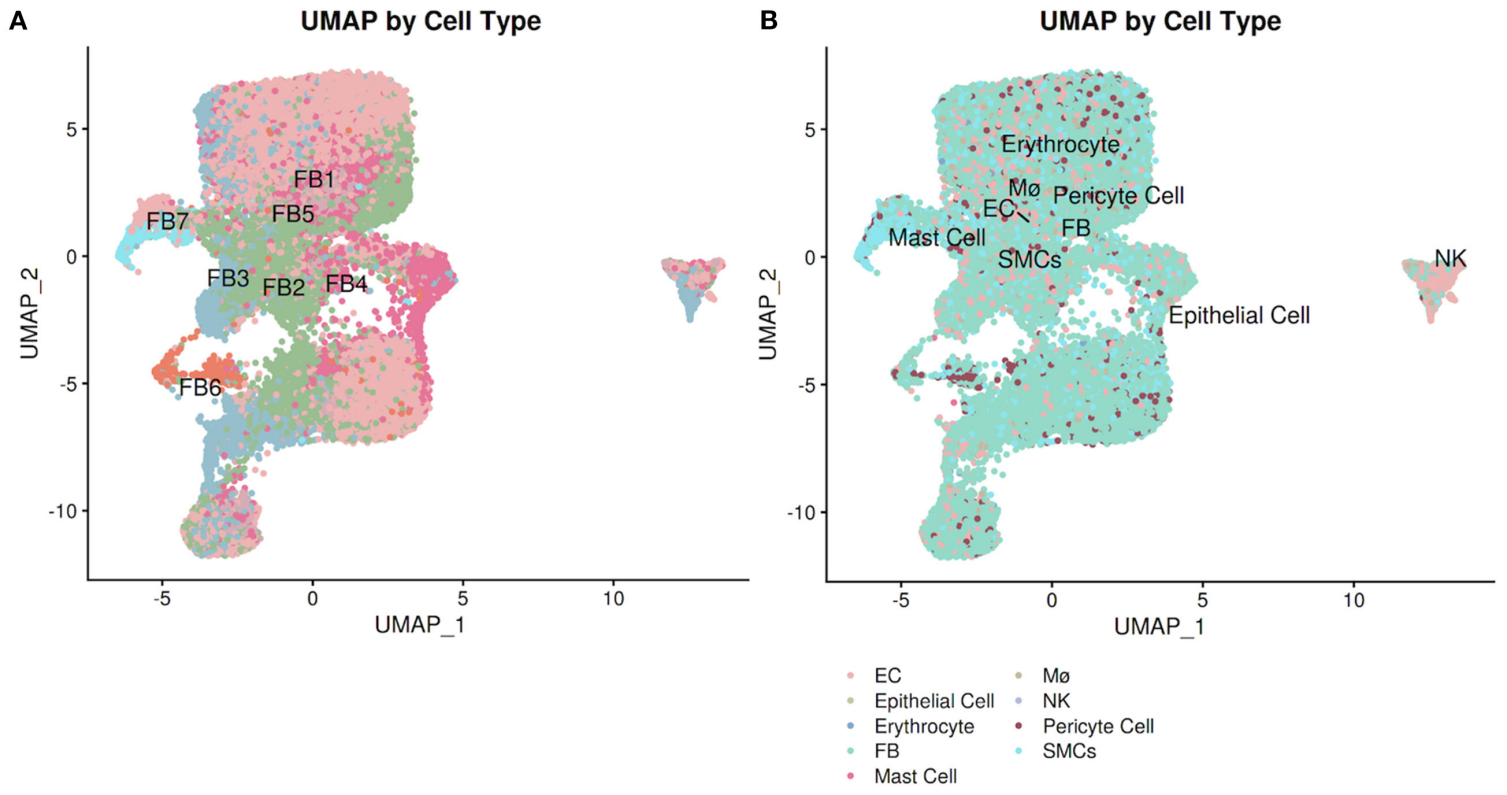
Annotation explorer page facilitates cell-level annotation comparison. Users can compare pre-computed cell identity annotations as well as author-provided labels (when available). Using the Litvinukova et al. dataset, we demonstrate the difference between the annotation from the **(A)** original authors and **(B)** Seurat label transfer using the Tabula Sapiens reference.

PlaqView enables users to run and compare several methods of cell annotation and compare them against other databases, as well as exporting the entire differential gene expression matrix for manual exploration of rare cell types. In many areas of the application and specifically under the “Cell Labeling” tab, users have the ability to explore the cell identities as provided by the original authors (when available), by SingleR (28), and by Seurat V4 “label transfer” using the Tabula Sapiens atlas (7, 29). To demonstrate this, we explored the Livinukova et al., annotation (Figure 4A) against the annotation provided by Seurat's label transfer using the Tabula Sapiens reference (Figure 4B) (7, 29). These identity predictions are pre-run during the data processing stage and stored within each data object. By running the cell identity prediction during the preprocessing stage (see Methods) and not on-demand, PlaqView can rapidly display results with little downtime. To allow additional flexibility, transparency and further exploration of the differentially expressed genes in each cell type, we provide precomputed tables of the differentially expressed genes based on labeling methods in downloadable .csv format. By precomputing these tables during the preprocessing stage, we cut down hours of computing time for the end-user. These tables provide users the opportunities to review genes or cell groups of interests, and serve as starting points for downstream analysis such as drug-gene interaction analysis.

**Figure 5:**
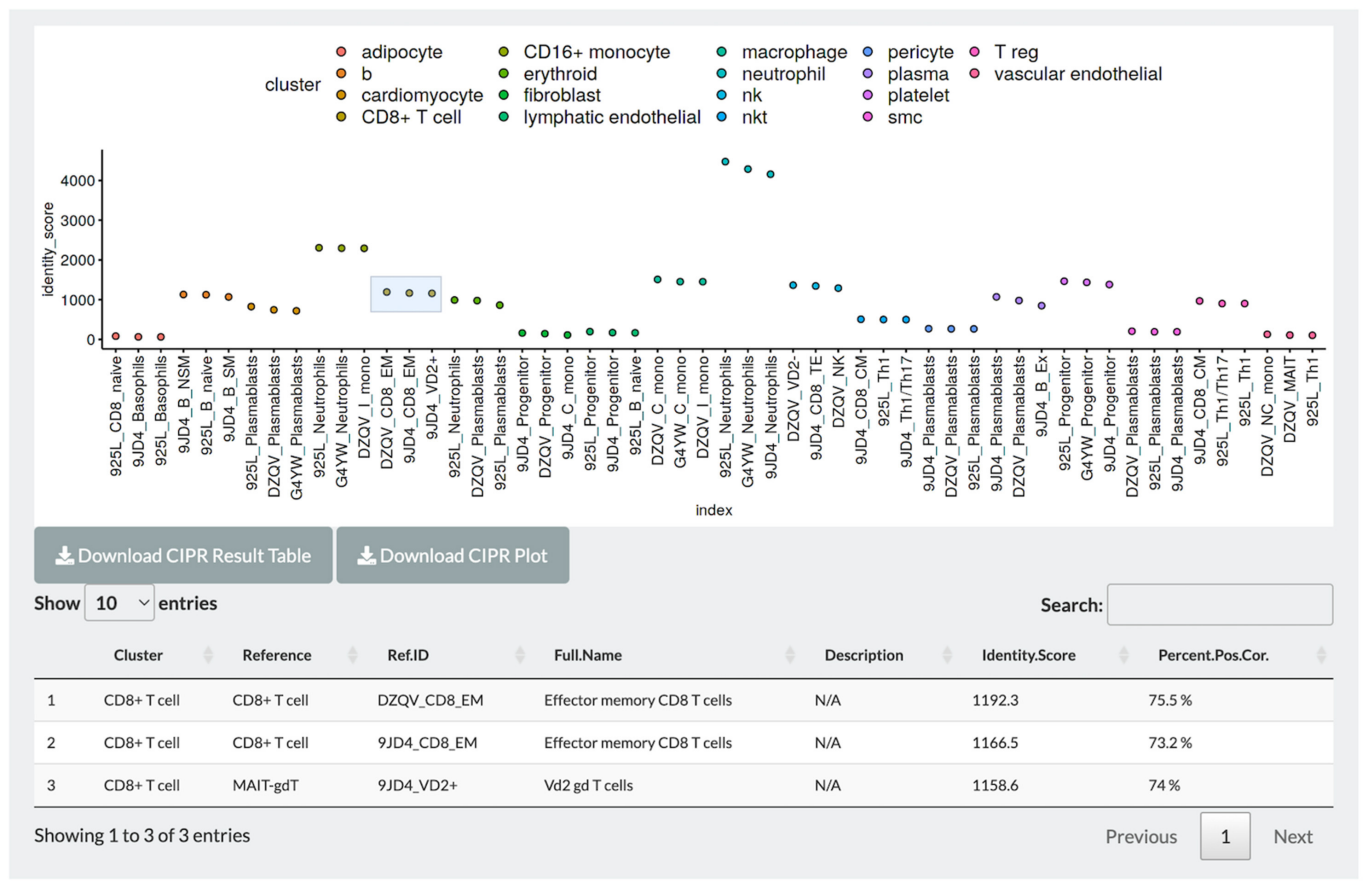
CIPR integration allows further exploration and interaction with cell-level annotation. Users can benchmark pre-computed annotations with existing single-cell references, and interactively compare and visualize identity scores and percent correlations with the top candidate reference identities. Light blue box indicates selected groups for detailed tables.

In PlaqView 2.0, we have incorporated a new interactive feature named Cell Identity PRedictor, or CIPR (Figure 5) (30). CIPR provides an additional opportunity for users to interact with the data and further explore and compare the cell annotations. CIPR calculates, in real-time, cluster-based gene expression similarity index scores against known references, such as the Database of Immune Cell Expression (DICE), Immunological GenomeProject (ImmGen), and the Human Primary Cell Atlas (30). To run CIPR on the loaded dataset, users need to select the starting labeling method (default is the unlabeled Seurat clusters) and the reference to benchmark against (default is ImmGen Mouse). CIPR was designed to be able to run against human and mouse references interchangeably. PlaqView will output an interactive CIPR plot where users can select the cluster(s) of interest and explore the top similar cell types, their descriptions, identity scores (calculated as a function of fold-change dot-product) and percent of genes that are similarly co-expressed. For example, when running the COVID-19 heart autopsy data from Delorey et al. (19) using the pre-sorted human RNAseq reference provided by CIPR, we noted a high concordance between the author-labeled “CD8 + *T*-cells” with the reference cluster “Effector memory CD8 *T* cells”, with a 75.5% precent positive correlation in gene signature (Figure 5). Lastly, users can download a full table of the CIPR results in .csv format as well as the CIPR plot in .pdf format for publication.

### PlaqView enables users to explore unstructured metadata

Currently, no systematic convention exists in sharing single-cell metadata that are essential for reproducible analysis and future meta-analysis. These important metadata, such as sex, age, sample location, and author-provided cell-type annotations, are often omitted when submitting data to public repositories. It is estimated that fewer than 25% of current single-cell studies have provided cell-level metadata (4). PlaqView 2.0 aims to provide a platform for easier standardization and sharing of cell-level metadata in three ways: (1) we curate and reformat existing metadata and append them into the Seurat object, (2) we require new submissions to have pre-embedded metadata, and (3) we developed features to explore all existing available metadata in their unabridged format.

**Figure 6:**
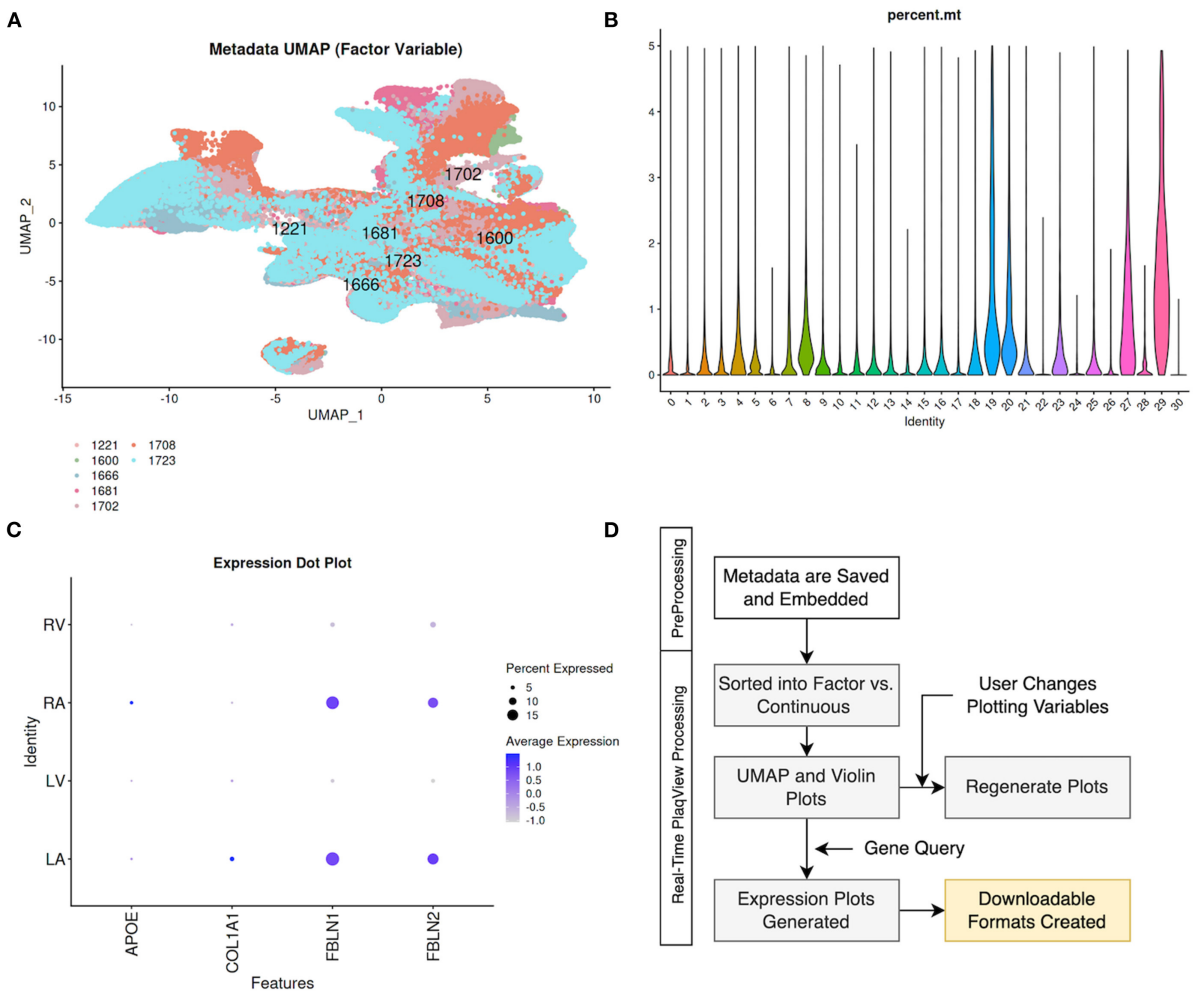
Metadata Explorer enables visualization and query of unabridged cell-level metadata. When available, cell-level metadata are divided into **(A)** factor-type such as cells separated by “biological_individuals,” and **(B)** continuous-type such as percent mitochondria by seurat cluster for visualization. **(C)** Users can also query gene expression based on factor-type metadata such as the heart “chamber” in the case of Tucker et al. (31). **(D)** We embed the metadata during the preprocessing stage and PlaqView sorts the metadata and runs calculations in response to the user selection to generate corresponding UMAPs and Violin plots.

When available, PlaqView separates the metadata into “Factor-Type” (Figure 6A) and “Continuous-Type” variables (Figures 6B,D). Examples of factor-type metadata include sex, age-group, biological individuals, and disease type, whereas continuous-type metadata include percent mitochondrial reads, age, and *p*-values of singleR annotations. PlaqView will output appropriate feature maps when these data are available. Furthermore, we introduce the ability to query gene expression based on factor-type variables. We demonstrate its use by querying the genes *APOE, COL1A1, FBLN1*, and *FBLN2* in the Tucker et al. (31) dataset using the “chamber” variable (Figure 6C). Interestingly, fibulins (*FBLN1* and *FBLN2*) are more highly expressed in the right atrium (RA) and left atrium (LA), compared to ventricular samples, most likely due to the differential behavior of atrial and ventricular fibroblasts (32). However, further interrogation using the “experiment” and “biological.individual” variables show significant variation of fibulin expression among the atrial samples as well as among individuals sampled (Supplemental Figures 1A,B). This particular example demonstrates the critical need for better metadata sharing as well as the utility of the metadata explorer feature.

### Cell trajectories and re-clustering

**Figure 7:**
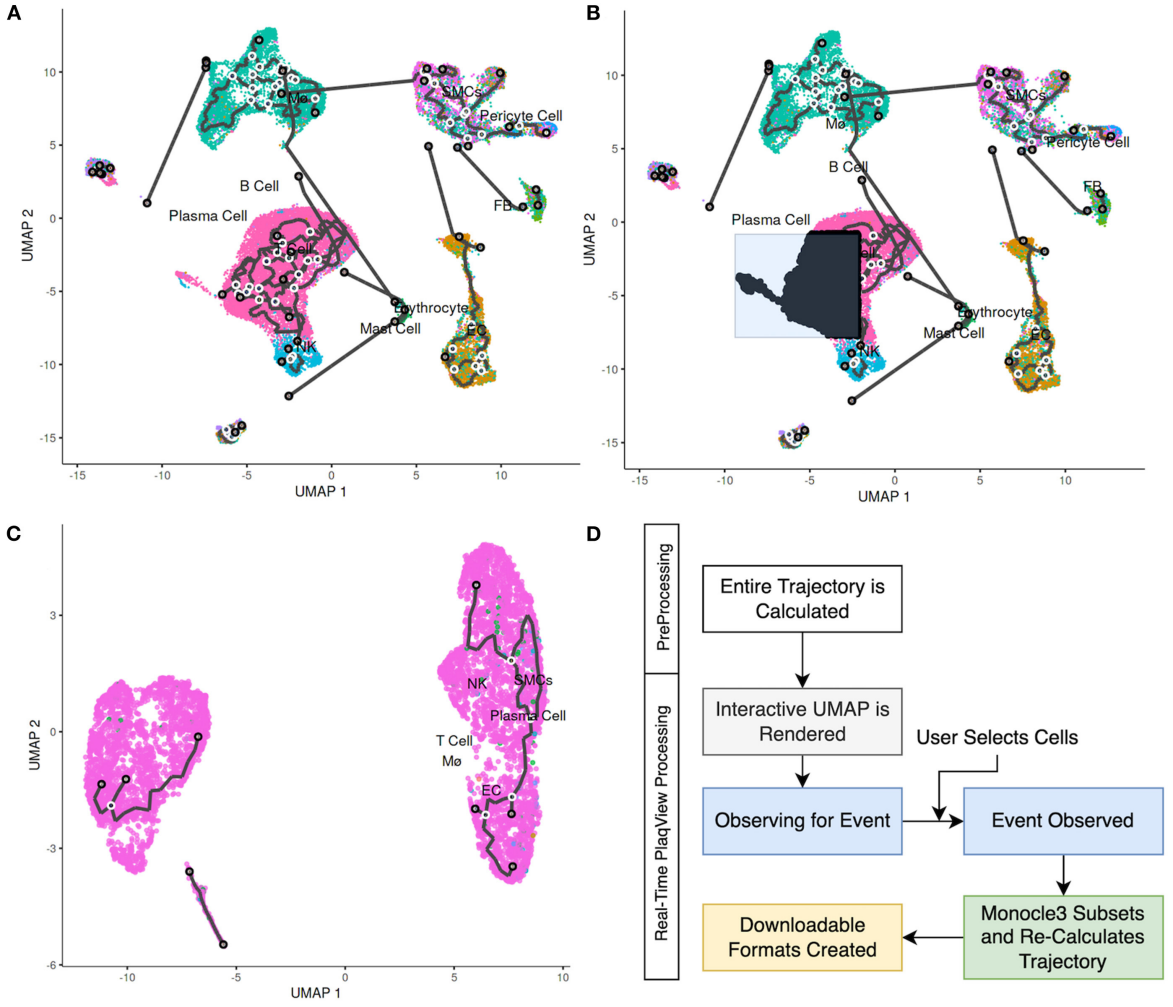
PlaqView 2.0 enables interactive RNA trajectory inference. **(A)** Full cell trajectory inferences are presented to the user, as demonstrated using the Alsaigh et al. dataset. **(B)** Selected cells are highlighted in black. These cells can be subsetted and their **(C)** trajectories can be re-calculated in PlaqView as powered by Monocle 3. **(D)** We pre-compute the overall trajectory during the pre-processing stage, and recalculations of subset trajectories are done *ad hoc* as needed by PlaqView as an on-demand function.

RNA trajectory analysis, in conjunction with pseudotemporal ordering, has been widely used as a method to reconstruct cell fate, differentiation, and transition events (12, 33–35). In terms of cardiovascular data, RNA trajectories have been useful in studying the fate and transitions of fibroblasts, immune cells, smooth muscle cells and other intermediate cell types (12, 35–37). In PlaqView 2.0, we provide additional functionality in RNA trajectory estimation with Monocle3 (33) by integrating high-performance calculation steps directly within the browser. Upon opening the “Trajectory” page, the full pre-calculated RNA trajectory using the entire dataset is displayed. Now, users can select cells of interest and subset these cells for re-clustering and re-calculation of their RNA-trajectory (Figures 7A-C). This interactive feature allows supervised input and selection of relevant cells that can help reduce trajectory “noise.” Furthermore, this feature is helpful because RNA trajectories may not always be applicable to every cell type in the study, such as those that are post-mitotic or slow-dividing. Depending on the number of selected cells, *ad hoc* calculation of RNA-trajectory can take up to 10–15 min, which is longer than a typical shiny application timeout rule (Figure 7D). We made custom services rules in the backend container to ensure that RNA-trajectories are calculated in the most time-efficient manner possible and modified any timeout rules typically applied to reliably deliver RNA-trajectory results. To handle larger datasets in the future, we will implement a notification and ticketing system to allow users to return to the same session when calculations are complete. This new interactive feature allows users to focus on their cell types of interest to better develop future hypotheses and experiments.

### Druggable genome and cell targeting

Lastly, to enable researchers to rapidly explore current drug databases in the context of relevant single-cell datasets, we integrated the Drug Gene Interaction Database (DGIdb v4) with PlaqView (38, 39). In the “Druggable Genome” tab, users can input a gene of interest to simultaneously query the gene expression within the loaded single-cell dataset as well as display potential drug interactions (Supplemental Figure 2). Now, users can select multiple databases including Catalog of Somatic Mutation in Cancer (COSMIC), Food and Drug Administration (FDA), and DrugBank as described in DGIdb v4 (40). Additionally, users can download the corresponding UMAP as .pdf as well as the full drug-interaction table as .csv formats. This feature is invaluable in rapidly formulating hypotheses and future drug-repurposing experiments.

### Goals and future updates

We are committed to bringing the most updated and relevant cardiovascular datasets to PlaqView. Currently, we plan to update the PlaqView database at least once monthly as more datasets and studies are published. Furthermore, we are currently developing other multi-modal portals suited for data such as single-cell Assay for Transposase-Accessible Chromatin (scATAC-seq), spatial RNA-seq, and a separate portal to compare healthy and diseased tissues in a systematic manner.

## Materials and methods

### Data formatting

One of the major challenges of reproducible single-cell analysis is the lack of standardization in data-sharing format. We have found that the most commonly used methods are: (1) sharing processed data as matrices (e.g., one file for counts, one file for metadata, etc.), (2) sharing raw FASTQ files, (3) sharing processed Seurat objects as .rds files or equivalent, such as .h5ad files. We have found that sharing single-cell data as .rds or equivalent is the most convenient and reproducible method as the metadata are matched at the cell-level. However, as noted above, fewer than 25% of published single-cell RNA-seq studies provide this cell-level metadata.

For PlaqView, each dataset curated or submitted is standardized and is ready to be read by the application. Although some efforts have been made to allow interconversions between file formats, such as sceasy (https://github.com/cellgeni/sceasy), manual effort is still required to standardize analysis input for PlaqView. Depending on the incoming file type, they are converted or updated into the latest Seurat object class in R, and are saved as .rds files. These processed files, along with their raw formats, are made publicly available on the PlaqView homepage at https://plaqview.com/data. Systematic processing script is available in in the PlaqView DataProcessing Github page (https://github.com/MillerLab-CPHG/PlaqView_DataProcessing) and can be used as a reference for other single-cell applications to readily convert datasets into R-readable formats.

### Data processing steps

**Figure 8:**
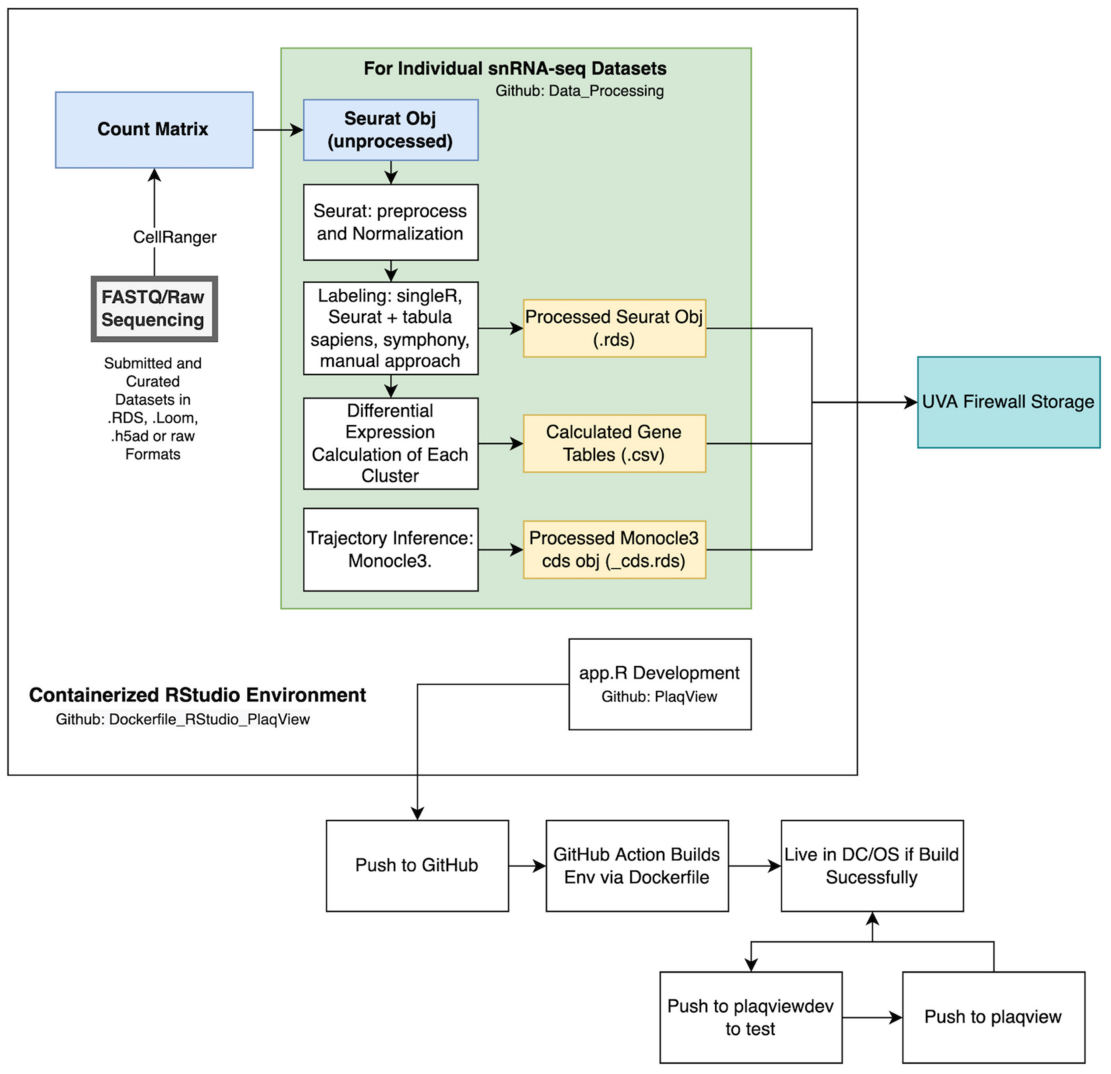
Overview of data processing and programmatic strategy for PlaqView. Data submitted to PlaqView are processed systematically and stored as Seurat.rds objects. These objects, along with calculated differential gene expression tables and trajectories, are stored in secured storage provided by the University of Virginia. Additionally, app development and data processes are all conducted in a cataloged, stable Docker RStudio environment that is registered both on GitHub and DockerHub.

Once the datasets are converted into the raw Seurat objects, we process them in the same manner to generate several output files to be read by PlaqView (Figure 8). First, we filter out low quality cells that have <200 or >2,500 features, and those with >5% mitochondrial reads. Exceptions are made for particular datasets that evaluate mitochondrial read data, such as data from Li et al. (13). Then, standard Seurat preprocessing using the following functions are conducted: FindVariableFeatures(), NormalizeData(), ScaleData(), RunPCA(), RunUMAP(), FindNeighbors(), and FindClusters().

To infer cell identity using automatic methods, the scaled RNA matrix is extracted using the GetAssayData() function and fed into SingleR as a new singleR object using the SingleR() function. The identities called by singleR are added into the metadata slot within the Seurat object.

Similarly, we use Seurat's label transfer function FindTransferAnchors() and TransferData() to predict cell identity using the Tabula Sapiens and Tabula Muris (41) references, depending on the original species. Identity calls are added to the metadata column using the AddMetadata() function. Some longer cell labels, such as “Smooth_muscle_cells” are shortened to “SMC.” The final Seurat object that contains the SingleR calls, Seurat calls, Seurat clusters (numbered clusters) are exported as an .rds file (Figure 8).

To enable trajectory analysis, we used custom scripts to extract Seurat data and place them into a Monocle3 CDS object using the new_cell_data_set() function. The new CDS object is preprocessed using the following functions: preprocess_cds(), reduce_dimension(), cluster_cells() (33, 34). A custom script is used to overlay the Seurat UMAP embedding into the Monocle3 object for consistent visualization. Then, starting nodes are selected automatically based on the closest vertex followed by learn_graph() and order_cells(). The resulting Monocle3 object is exported as an _cds.rds file (Figure 8).

Finally, we compute the entire differential expression for all cell types labeled by different methods (i.e., Seurat clusters, author-provided, singleR, and Seurat/Tabula Sapiens annotations) using the FindAllMarkers() function. This is the most time-consuming and memory intensive part of the preprocessing pipeline and we use the Future package to parallelize this step. The full R script for the preprocessing pipeline is located in our Data Processing Github page (https://github.com/MillerLab-CPHG/PlaqView_DataProcessing).

### Data storage, submission and requests

Raw human sequencing data often require specialized secured storage both due to their size and institutional review board (IRB) compliance regulations. Currently, PlaqView 2.0 only requires the downstream count matrices and does not use any raw sequencing. When data are submitted to PlaqView as raw sequences (such as FASTQ or BAM files), they are processed offline in a dedicated high-performance computing platform and only the count matrices are transferred to PlaqView storage, which is protected under an institutional firewall (Figure 8).

Nonetheless, most datasets currently on PlaqView are already open-access and are deposited in different public storage spaces in various raw formats. All datasets available on PlaqView have undergone systematic preprocessing and are saved in .rds formats that can be requested directly on PlaqView.com under “Data.” Furthermore, researchers can directly and securely submit their dataset to PlaqView on the PlaqView homepage.

### Reproducible computing environment

Various approaches in computer science and within the R community have been used to create reproducible and stable computing environments to facilitate faster new user setup, scalability for larger datasets, and increased stability of web applications. To our knowledge, popular built-in tools such as *renv* does not completely enable a reproducible environment and only records the versions of tools in an R computing environment (https://rstudio.github.io/renv/articles/renv.html). Recently, the combination of Docker and R gave rise to the Rocker Project (https://www.rocker-project.org/), which utilizes the image building capability of Docker in conjunction with R. Essentially, a predetermined set of R tools are installed on top of a basic operating system, such as Ubuntu. This enables programmers to capture the entire computing environment- including the base operating system, R, and all dependent packages- and can be downloaded to any computing platform reliably and in isolation. This has an advantage over other tools such as *renv* in that Docker images contain the actual packages and operating system, therefore in the event of version changes or deleted repositories, it will not break with the computing environment. Based on Rocker, we have built a custom Docker image that enables users to run the exact analysis pipelines with all dependencies already installed, and is available *via* DockerHub at millerlab/plaqviewmaster. This Docker image has two major utilizations: as a stable environment for feature development and as a base for the PlaqView application deployment, as it contains all the necessary packages and serves as a backbone for the web application (Figure 8).

### Service structure overview

PlaqView is designed to enable researchers to interact with high-performance computing *via* the web browser. The superstructure of PlaqView is to translate user input and selection from the browser *via* Shiny and run calculations in R, which runs in a clustered container orchestration environment alongside the University of Virginia High Performance Computing system (Supplemental Figure 3A). To enable user interactions with the data without coding knowledge, each interactive element in PlaqView is coded as Shiny Reactive elements, which change the underlying R code snippets in preparation for the analysis. For example, when a user selects a dataset, the corresponding values of the working directory is changed to the selected value, and when the user clicks “Load Dataset”, Shiny monitors and triggers the event to execute the R code to load the .rds files within the working directories.

Currently, we are using DC/OS (Distributed Cloud Operating System) to regulate the amount of memory and processor each user can access. DC/OS also automatically handles the workload demand to scale up more service instances in response to increased user access. Furthermore, this infrastructure allows for isolated, or “sticky,” instances so each user is given a dedicated R instance and cannot access other users' instances. This is commonly referred to as “stateless programming.” Currently, PlaqView supports up to 32GB of memory per user (the memory required to access the largest dataset, and can be scaled up as needed). In the near future, we plan to further scale up using Kubernetes on large commercial-grade infrastructures such as Google Cloud Computing.

### Development workflow

Typically, PlaqView development occurs in several stages: edit source code (app.r file), test locally and update Docker images, deployment, and bug fixes (Supplemental Figure 3B). The standard development workflow starts with editing the app.R script, which encompasses the UI (user interface) and the Server codes (codes that calculate and compute results). New features and codes are tested locally in the Dockerized RStudio container for bugs. Final edits are pushed to GitHub as “commits” and new changes initiate GitHub Actions to recompile the application from the base Docker image. Normally, this step involves reinstallation of the base operating Linux system and its dependencies, R, Shiny, and all dependent R packages from scratch, and typically takes about 60 min. However, at this stage, we have simplified the building process by pulling the aforementioned pre-built Docker base image. In our experience, the typical rebuild takes about 60 min whereas pulling the stable base Docker image from DockerHub takes only 3–4 min.

Once the app is live, we begin to capture user feedback and fix any additional bugs. We implement bug fixes and feature requests *via* GitHub as well as through internal runtime log reviews.

### Alternative service structure and adaptation to other fields of research

PlaqView was originally developed for atherosclerosis-related cardiovascular datasets, but the underlying structure was designed to be easily adapted to other fields of research. The entirety of the source-code has been made available on GitHub. Furthermore, each iteration of PlaqView comes with a containerized base image hosted on DockerHub (wfma888/plaqviewmaster), which allows for immediate and reproducible deployment in virtually any computing structure. Essentially, there are two major steps to adapt PlaqView to other fields: minimal processing of the user interface script and deploying to a suitable service structure.

Due to the size of the single-cell datasets, PlaqView and adapted versions are best hosted on dedicated, large high-performance clusters. Commercial solutions that would work well are Google Cloud Services as Google Run instances or Amazon Web Services. Shinyapps.io also provides a native and easy way to deploy Shiny apps for beta testing, however limitations in memory per instance and slow performance limits its usefulness in analyzing large datasets.

## Discussion

To date, very few single-cell portals like PlaqView exist for cardiovascular genomics research. ExpressHeart, a single-cell portal dedicated to non-cardiomyocyte cells, has several single-cell datasets but has limited scope and features for data re-analysis (Supplemental Table 1) (42). Other, larger data portals such as the Broad Single Cell Portal (SCP) have many large studies but failed to include many critical cardiovascular datasets such as Wirka et al. (11) and Xu et al. (15), and lack the focus on cardiovascular diseases in general. PlaqView aims to bridge the gap between large, multi-organ portals like SCP and niche portals such as ExpressHeart and serve as a critical resource for the cardiovascular field.

PlaqView helps to overcome many modern challenges in the single-cell field, such as the complex coding and computational knowledge needed to explore single-cell data and standardization for data sharing. Since its initial release, we have registered users from 35 countries, with the top being U.S., China, Germany, and the Netherlands. To our knowledge, PlaqView is the most comprehensive single-cell portal dedicated to cardiovascular research. We are committed to the longevity of PlaqView and are working on furthering PlaqView's capability as multimodal datasets are released, such as spatial and scATAC-seq data. Our immediate goals are (1) including additional relevant single-cell datasets, (2) creating a subportal for live single-cell dataset integration and comparison, and (3) creating a subportal for multimodal single-cell data visualization.

### Contribution to the cardiovascular field

Single-cell data has always been challenging to share, analyze, and visualize. Typically, these datasets require specialized computing knowledge and high-performance computing tools not readily available. Previously, we presented PlaqView, a web-portal to allow lay scientists and benchtop researchers to rapidly view single-cell RNA-seq data for atherosclerosis. Here, we introduce PlaqView 2.0, a significant improvement to the PlaqView application. In this second major release, we introduce many new features, such as a metadata explorer, cell identity prediction, and *ad hoc* RNA-trajectory calculations. We further improved the usability, speed, stability, and scale of the application. PlaqView serves both as a repository of single-cell data for cardiovascular diseases as well as a tool to rapidly visualize, renalayze, and share scRNA-seq data without the need to code or have specialized computing knowledge. PlaqView is an invaluable resource that bridges the gap between computational and experimental research to advance cardiovascular medicine.

## Data availability statement

Publicly available datasets were analyzed in this study. This data can be found here: www.plaqview.com.

## Ethics statement

The studies involving human participants were reviewed and approved by Institutional Review Board (IRB) at the University of Virginia. The patients/participants provided their written informed consent to participate in this study.

## Author contributions

WFM designed, tested, and wrote the PlaqView application. AWT provided feedback, programming help, and application testing. CG provided back-end infrastructure and deployment aid. DW, YS, JVM, GA, CJH, and AP provided feedback and application testing. SWvL and CLM guided and helped in the development of PlaqView. HAE developed the CIPR module. All authors provided feedback and editing for this manuscript.

## Funding

Funding for this research was provided by National Institutes of Health (NIH) grants R00HL125912 and R01HL14823, a Leducq Foundation Transatlantic Network of Excellence (PlaqOmics) Young Investigator Grant, Netherlands CardioVascular Research Initiative of the Netherlands Heart Foundation (CVON 2011/B019 and CVON 2017-20: Generating the best evidence-based pharmaceutical targets for atherosclerosis [GENIUS I&II]), and the ERA-CVD program druggable-MI-targets (grant number: 01KL1802). SL was funded through EU H2020 TO_AITION (grant number: 848146).

## Conflict of interest

Author SL has received Roche funding for unrelated work. The remining authors declare that the research was conducted in the absence of any commercial or financial relationships that could be construed as a potential conflict of interest.

## Publisher's note

All claims expressed in this article are solely those of the authors and do not necessarily represent those of their affiliated organizations, or those of the publisher, the editors and the reviewers. Any product that may be evaluated in this article, or claim that may be made by its manufacturer, is not guaranteed or endorsed by the publisher.

## Abbreviations

CIPR: cell identity predictor
GSEA: gene-set enrichment analysis
scRNA-seq: single-cell RNA-sequencing
UMAP: uniform manifold approximation and projection.
