# Supplementary material for: PlaqView 2.0: A comprehensive web portal for cardiovascular single-cell genomics
Source: Front Cardiovasc Med. 2022 Aug 8;9:969421. doi: 10.3389/fcvm.2022.969421 (PMC9393487; doi:10.3389/fcvm.2022.969421)
Supplement: Supplementary file 1 [file Table_1.DOCX]

**Supplemental Table 1.**

| **Database** | **scRNA-seq Focus?** | **Cardiovascular Focus** | **Gene Query** | **Metadata Explorer** | **Drug-Gene Interaction** | **RNA-Trajectory** | **Data Available for Download** | **Link** | **Reference** |
| --- | --- | --- | --- | --- | --- | --- | --- | --- | --- |
| Broad Institute Single Cell Portal | Yes | Some, but mostly general purpose single-cell database | Yes | Yes | No | No | Yes | <https://singlecell.broadinstitute.org/single_cell> | NA |
| CLARA | Yes | Yes, currently 6 datasets | Yes | No | No | No | Some | <https://pinto-lab.shinyapps.io/clara/> | [(1–3)](https://app.readcube.com/library/e6ddebf6-a90f-485d-a131-51d7cb167bd7/all?uuid=07951025083860919&item_ids=e6ddebf6-a90f-485d-a131-51d7cb167bd7:c46a545a-cd55-4536-a049-88fe123f7398,e6ddebf6-a90f-485d-a131-51d7cb167bd7:7beb8ec6-5efc-45de-8848-d1abf8f2fb6d,e6ddebf6-a90f-485d-a131-51d7cb167bd7:3c915ca5-b4d4-4b1b-bf20-4e8691475453) |
| UCSC Cell Browser | Yes | Some, but mostly general purpose single-cell database | Yes | Yes | No | No | Most | <http://cells.ucsc.edu/?> | [(4)](https://app.readcube.com/library/e6ddebf6-a90f-485d-a131-51d7cb167bd7/all?uuid=35054167304036654&item_ids=e6ddebf6-a90f-485d-a131-51d7cb167bd7:c0f5f374-25a9-478a-9247-70404a68da84) |
| CARE Portal | Yes | Only one study, based on UCSC | Yes | Yes | No | No | Yes | <http://ns104190.ip-147-135-44.us/data_CARE_portal/snATAC/ucsc_browser/> | [(2)](https://app.readcube.com/library/e6ddebf6-a90f-485d-a131-51d7cb167bd7/all?uuid=7119385725052363&item_ids=e6ddebf6-a90f-485d-a131-51d7cb167bd7:7beb8ec6-5efc-45de-8848-d1abf8f2fb6d) |
| Express Heart | Yes | Yes, currently 5 datasets | Yes | Yes | No | No | No | <http://shiny.bios.unc.edu/expressheart/> | [(1,2)](https://app.readcube.com/library/e6ddebf6-a90f-485d-a131-51d7cb167bd7/all?uuid=03812609261581856&item_ids=e6ddebf6-a90f-485d-a131-51d7cb167bd7:c46a545a-cd55-4536-a049-88fe123f7398,e6ddebf6-a90f-485d-a131-51d7cb167bd7:7beb8ec6-5efc-45de-8848-d1abf8f2fb6d) |
| Heart Cell Atlas | Yes | Only one study, based on UCSC | Yes | Yes | No | No | Yes | <https://www.heartcellatlas.org/> | [(2,5)](https://app.readcube.com/library/e6ddebf6-a90f-485d-a131-51d7cb167bd7/all?uuid=17954650167749453&item_ids=e6ddebf6-a90f-485d-a131-51d7cb167bd7:7beb8ec6-5efc-45de-8848-d1abf8f2fb6d,e6ddebf6-a90f-485d-a131-51d7cb167bd7:c13f5614-fc62-4e05-813d-75e8609178bc) |
| HeartBioPortal | No, GWAS focused | Yes | Yes | No | No | No | No | <https://www.heartbioportal.com/> | [(6–8)](https://app.readcube.com/library/e6ddebf6-a90f-485d-a131-51d7cb167bd7/all?uuid=48972538915743713&item_ids=e6ddebf6-a90f-485d-a131-51d7cb167bd7:30ebdbec-b995-430c-9e87-763bee1edf4c,e6ddebf6-a90f-485d-a131-51d7cb167bd7:f25cd855-661e-412e-9f2b-3d52a37db9e8,e6ddebf6-a90f-485d-a131-51d7cb167bd7:e0b47b2e-4c18-443b-ae79-91eb3c39158b) |
| cBioportal | No, bulk-RNA focused | No, cancer focused | Yes | No | No | No | No | <https://www.cbioportal.org/> | [(9)](https://app.readcube.com/library/e6ddebf6-a90f-485d-a131-51d7cb167bd7/all?uuid=25520495834138246&item_ids=e6ddebf6-a90f-485d-a131-51d7cb167bd7:9c47a858-32ba-4d6e-ad99-48423c77eac6) |
| DISCO | Yes | None | Yes | Yes | No | No | Some | https://www.immunesinglecell.org/ | [(1)](https://app.readcube.com/library/e6ddebf6-a90f-485d-a131-51d7cb167bd7/all?uuid=2297815520395754&item_ids=e6ddebf6-a90f-485d-a131-51d7cb167bd7:c46a545a-cd55-4536-a049-88fe123f7398) |
| **PlaqView** | **Yes** | **Yes** | **Yes** | **Yes** | **Yes** | **Yes** | **Yes** | **plaqview.com** | **NA** |

**References**

[1. Li M, Zhang X, Ang KS, Ling J, Sethi R, Lee NYS, Ginhoux F, Chen J. DISCO: a database of Deeply Integrated human Single-Cell Omics data. *Nucleic Acids Res* (2021) 50:gkab1020-. doi: 10.1093/nar/gkab1020
2. Li G, Luan C, Dong Y, Xie Y, Zentz SC, Zelt R, Roach J, Liu J, Qian L, Li Y, et al. ExpressHeart: Web Portal to Visualize Transcriptome Profiles of Non-Cardiomyocyte Cells. *Int J Mol Sci* (2021) 22:8943. doi: 10.3390/ijms22168943
3. Dona MSI, Hsu I, Rathnayake TS, Farrugia GE, Gaynor TL, Kharbanda M, Skelly DA, Pinto AR. CLARA: A web portal for interactive exploration of the cardiovascular cellular landscape in health and disease. *Biorxiv* (2021)2021.07.18.452862. doi: 10.1101/2021.07.18.452862
4. Speir ML, Bhaduri A, Markov NS, Moreno P, Nowakowski TJ, Papatheodorou I, Pollen AA, Raney BJ, Seninge L, Kent WJ, et al. UCSC Cell Browser: Visualize Your Single-Cell Data. *Bioinformatics* (2021) 37:btab503-. doi: 10.1093/bioinformatics/btab503
5. Litviňuková M, Talavera-López C, Maatz H, Reichart D, Worth CL, Lindberg EL, Kanda M, Polanski K, Heinig M, Lee M, et al. Cells of the adult human heart. *Nature* (2020)1–10. doi: 10.1038/s41586-020-2797-4
6. Khomtchouk BB, Tran D-T, Vand KA, Might M, Gozani O, Assimes TL. Cardioinformatics: the nexus of bioinformatics and precision cardiology. *Brief Bioinform* (2019) 21:2031–2051. doi: 10.1093/bib/bbz119
7. Khomtchouk BB, Vand KA, Koehler WC, Tran D-T, Middlebrook K, Sudhakaran S, Nelson CS, Gozani O, Assimes TL. HeartBioPortal: An Internet-of-Omics for Human Cardiovascular Disease Data. *Circulation Genom Precis Medicine* (2019) 12:e002426. doi: 10.1161/circgen.118.002426
8. Khomtchouk BB, Nelson CS, Vand KA, Palmisano S, Grossman RL. HeartBioPortal2.0: new developments and updates for genetic ancestry and cardiometabolic quantitative traits in diverse human populations. *Database* (2020) 2020:baaa115-. doi: 10.1093/database/baaa115
9. Gao J, Aksoy BA, Dogrusoz U, Dresdner G, Gross B, Sumer SO, Sun Y, Jacobsen A, Sinha R, Larsson E, et al. Integrative Analysis of Complex Cancer Genomics and Clinical Profiles Using the cBioPortal. *Sci Signal* (2013) 6:pl1–pl1. doi: 10.1126/scisignal.2004088](https://app.readcube.com/library/?style=Frontiers%20in%20Cardiovascular%20Medicine)
