# Supplementary figures and images for: PlaqView 2.0: A comprehensive web portal for cardiovascular single-cell genomics
Source: Front Cardiovasc Med. 2022 Aug 8;9:969421. doi: 10.3389/fcvm.2022.969421 (PMC9393487; doi:10.3389/fcvm.2022.969421)

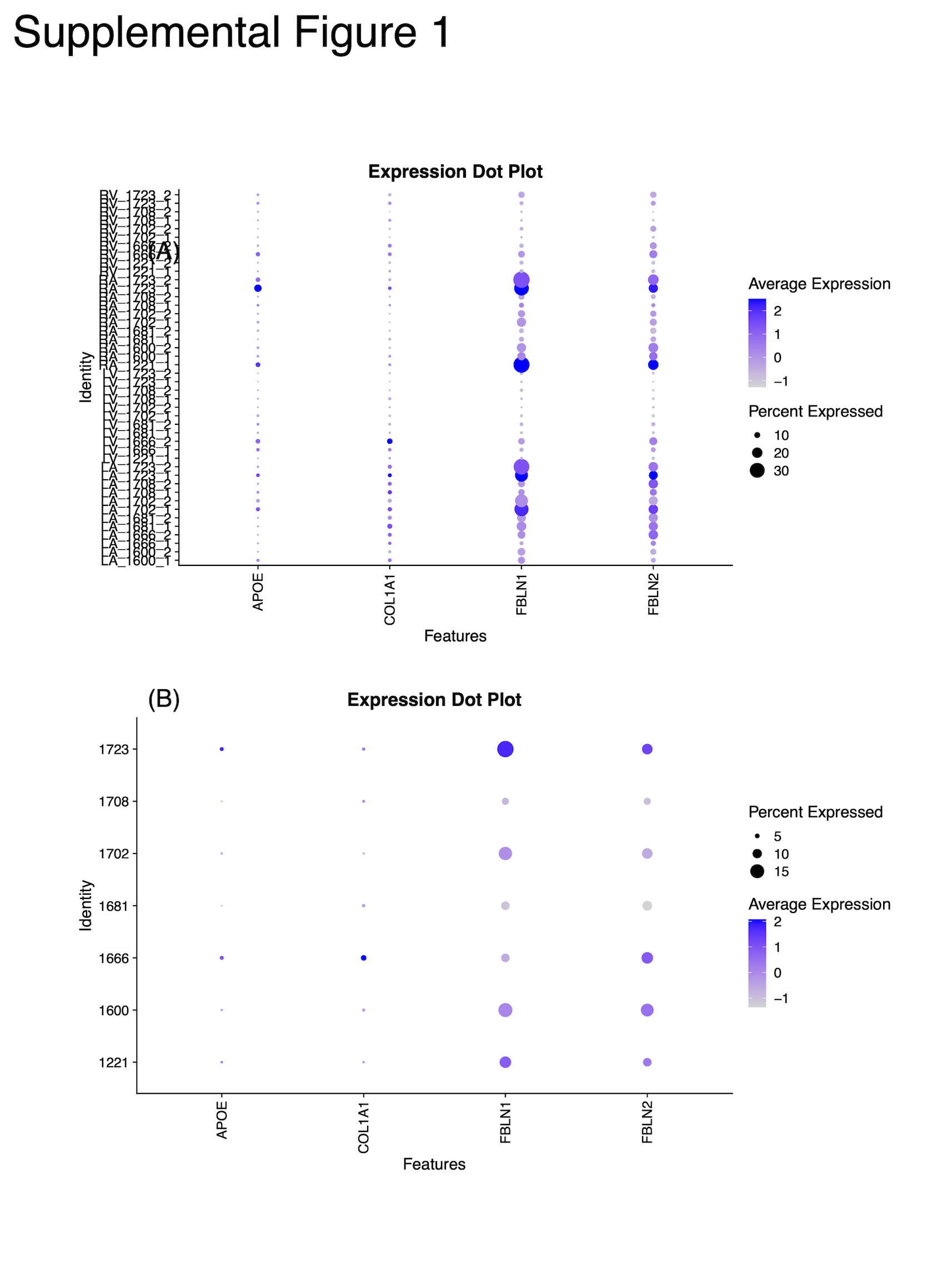

Supplement: Supplementary file 3 [file Image_1.TIFF]

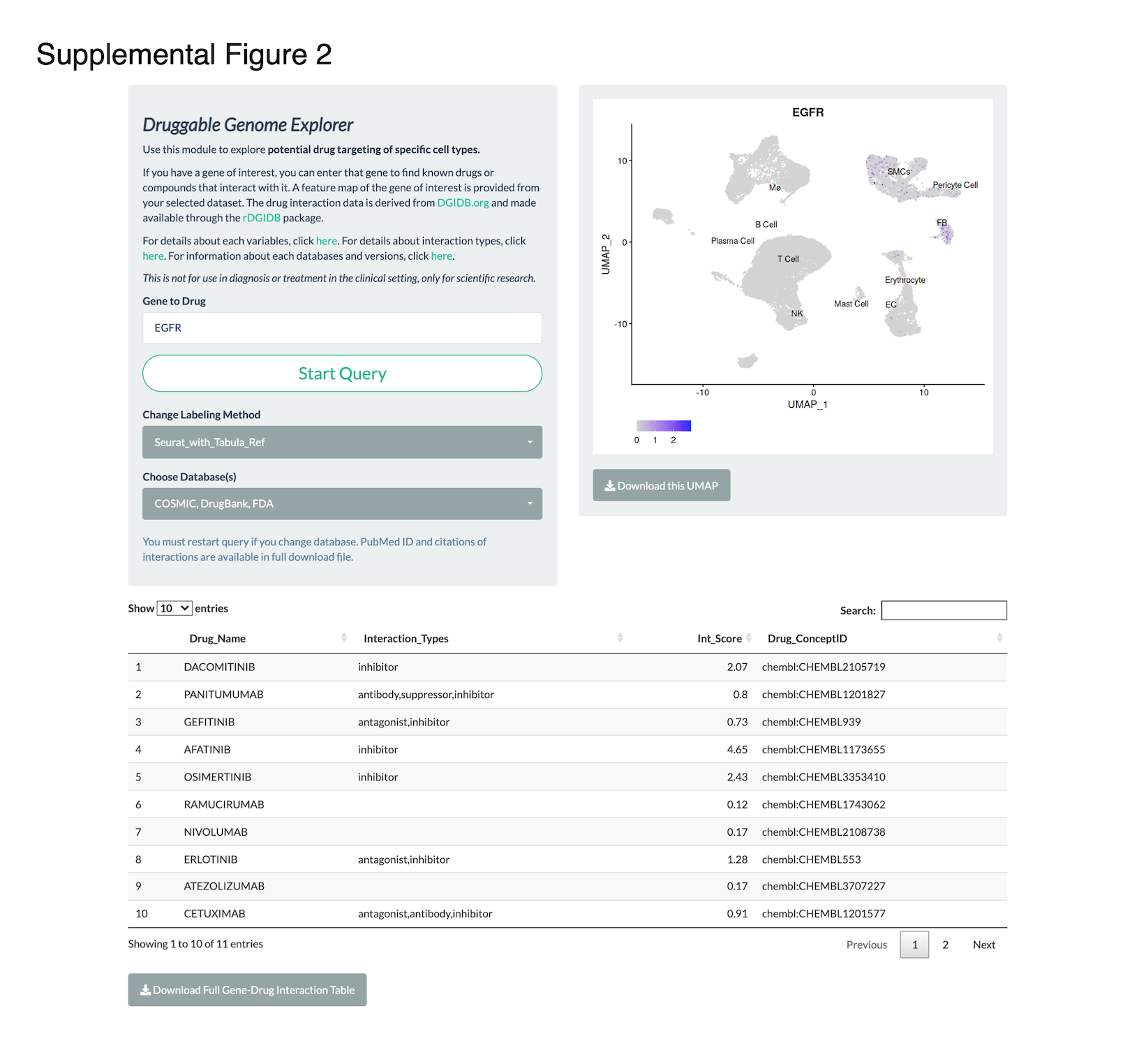

Supplement: Supplementary file 4 [file Image_2.TIFF]

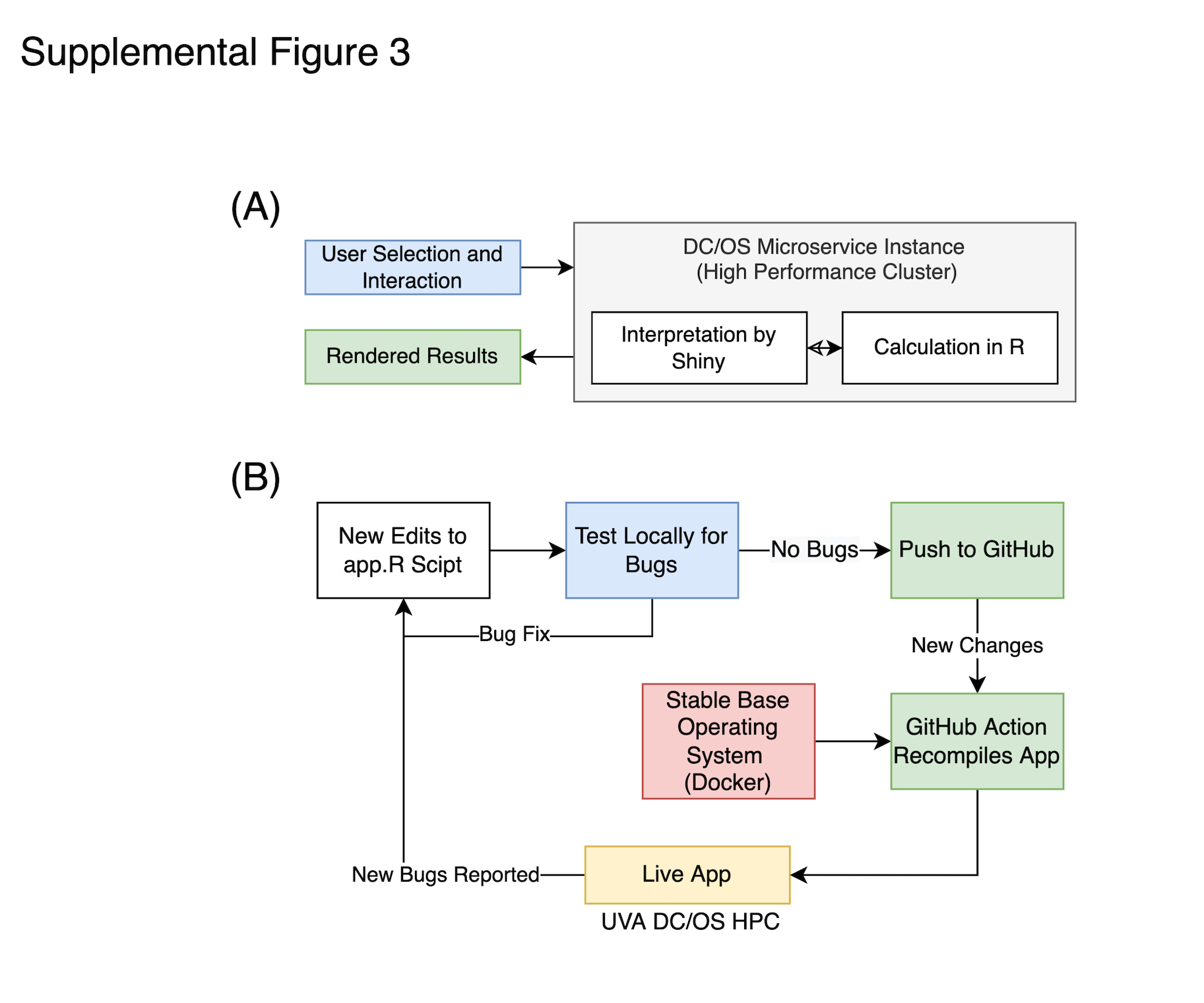

Supplement: Supplementary file 5 [file Image_3.TIFF]
